# Dynamics of pyrethroid resistance in malaria vectors in southern Benin following a large scale implementation of vector control interventions

**DOI:** 10.1186/s13071-016-1661-8

**Published:** 2016-07-04

**Authors:** Gildas A. Yahouédo, Sylvie Cornelie, Innocent Djègbè, Justine Ahlonsou, Sidick Aboubakar, Christophe Soares, Martin Akogbéto, Vincent Corbel

**Affiliations:** Institut de Recherche pour le Développement (IRD), Maladies Infectieuses et Vecteurs: Ecologie, Génétique, Evolution et Contrôle (MIVEGEC), UMR UM1-UM2 - CNRS 5290 – IRD 224, Montpellier, France; Centre de Recherche Entomologique de Cotonou (CREC), Cotonou, Bénin; Department of Entomology, Faculty of Agriculture, Kasetsart University, 50 Ngam Wong Wan Rd, Lat Yao Chatuchak, Bangkok, 10900 Thailand

**Keywords:** Malaria, Vector control, *Anopheles*, Insecticide resistance, *kdr*, Metabolic gene

## Abstract

**Background:**

Large-scale implementation of Indoor Residual Spraying and Insecticide Treated Nets has been implemented in Plateau Department, Benin between 2011 and 2014. The purpose of this study was to monitor the frequency and mechanisms of pyrethroid resistance in malaria vectors following the implementation of vector control tools for malaria prevention.

**Methods:**

*Anopheles* larvae were collected in 13 villages twice a year from 2012 to 2014. WHO tube tests were used to assess the phenotypic resistance of each population to 0.05 % deltamethrin. Sibling species within *Anopheles gambiae* complex were identified by PCR techniques. Taqman and biochemical assays were performed to identify the presence of *kdr* mutations in individual mosquitoes and to detect any increase in the activity of enzymes putatively involved in insecticide metabolism (oxidases, esterase and glutathione-*S*-transferases). Quantitative real time PCR was used to measure the expression of three metabolic genes involved in pyrethroid resistance (CYP6P3, CYP6M2 and GSTD3).

**Results:**

*Anopheles* populations showed < 90 % mortality to deltamethrin in all villages and at all time points. The *1014 F kdr* allele frequency was close to fixation (> 0.9) over the sampling periods in both *An. gambiae* and *An. coluzzii*. Biochemical assays showed higher activities of alpha esterase and GST in field malaria vector populations compared to susceptible mosquitoes. qPCR assays showed a significant increase of CYP6P3, CYP6M2 GSTD3 expression in *An. gambiae* after a three-year implementation of LLINs.

**Conclusion:**

The study confirmed that deltamethrin resistance is widespread in malaria vectors in Southern Benin. We suspect that the increase in deltamethrin resistance between 2012 and 2014 resulted from an increased expression of metabolic detoxification genes (CYP6M2 and CYP6P3) rather than from *kdr* mutations. It is urgent to evaluate further the impact of metabolic resistance on the efficacy of vector control interventions using pyrethroid insecticides.

## Background

Global malaria vector control efforts rely on the use of Long Lasting Insecticide Nets (LLINs) and Indoor Residual Spraying (IRS). Twelve insecticides belonging to four chemical classes (organochlorines, organophosphates, carbamates and pyrethroids) are approved by the World Health Organization (WHO) for malaria vector control. All of these insecticides are neurotoxic and either target acetyl cholinesterase in the synapses or the voltage-gated sodium channel (VGSC). Pyrethroids are the only insecticides recommended by the WHO for LLINs because of their low mammalian toxicity, fast action, and high insecticidal activity [1]. Unfortunately, pyrethroid resistance has developed in most malaria vector species worldwide including Africa [2]. Indeed, two major mechanisms are known to confer pyrethroid resistance in malaria vectors: target site modification (*kdr* mutations) and increased metabolism of insecticides through detoxifying enzymes. The *L1014F* and *L1014S* substitutions in the para VGSC in the domains III-IV are known to decrease affinity of pyrethroids for this receptor [3]. Recently the mutation *N1575Y* has been described to potentiate the effect of the *L1014F* mutation [4]. The second resistance mechanism is called “metabolic” through higher catalytic properties and/or overexpression of carboxylesterases (COEs), cytochrome P450 mono-oxygenases (P450s) and Glutathione S-Transferases (GSTs) [5]. Some members of these families such as CYP6M2, CYP6Z2, CYP6P3 are known to contribute to pyrethroid detoxification in *Anopheles* mosquitoes [6]. Both metabolic and target site (*kdr*) resistance are present west Africa [7] and particularly Benin [8] and are suspected to reduce the efficacy of vector control intervention [9, 10].

Since 2007 the National Malaria Control Program (NMCP) in Benin has implemented a nationwide distribution of LLINs. In 2011, 5,135,942 LLINs were distributed in Benin [11] followed by 5,663,220 in 2014 with a coverage of 97 % and 80 % respectively. In the study area, LLINs distributed in 2011 contained permethrin/deltamethrin (pyrethroids) while those distributed in 2014 contained deltamethrin only (data from NMCP). After implementation of vector control interventions, it is essential to monitor any changes in susceptibility/resistance status of malaria vectors populations to public health insecticide (WHO 2015). Many cross-sectional studies were done to address the spatial distribution of pyrethroid resistance in *Anopheles* at a given time [12] but few longitudinal studies were conducted to address temporal changes in pyrethroid resistance phenotype following the implementation of vector control interventions.

The aim of this study was to investigate the dynamic of pyrethroid resistance in malaria vectors from 2012 to 2014 in 13 villages of Plateau Department and to characterize the mechanisms involved by monitoring changes in the frequency and expression of pyrethroid-resistance markers. The outcomes shall help the NMCP to implement more effective vector control strategy against pyrethroid resistance populations.

## Methods

### Study area

The thirteen villages, selected on the basis of entomological and epidemiological criteria, were located in southeast Benin. They belonged to four districts: Ifangni, Sakete, Pobe, Ketou and were visited for mosquito collection (Table 1). The study area is characterised by two rainy seasons from April to July and from September to October. The area is 3264 square km with a total population of 407,116 inhabitants (General Census of Population and Housing, 2002). Inhabitants of these villages are mostly farmers, traders, gardeners and fish breeders. Farmers grow cereals (maize, groundnuts, and beans), tubers (yams, manioc) and some vegetable crops such as tomato (Ifangni), chilli (Ketou). The fish breeding was only conducted in Itassoumba village (Ifangni) where tilapias and catfish were bred in large fishponds. These ponds provide a permanent breeding habitat for mosquitoes.

**Table 1:** Localisation and demographic information of study area

	Coordinates	Area (km^2^)	Population
Ifangni	6°40'N, 2°43'E	242	71,606
Sakete	6°44'N, 2°39'E	432	70,604
Pobe	6°58'N, 2°39'E	400	82,910
Ketou	7°21'N, 2°36'E	2183	100,499

### Mosquito collection

Mosquito collections were conducted during five rainy seasons. The larval sampling periods were June to August 2012 (termed June 2012), October to November 2012 (termed October 2012), May to July 2013 (termed June 2013), October to November 2013 (termed October 2013) and June to August 2014 (termed June 2014). In every survey, mosquito larvae were collected across the villages from several temporary breeding habitats, including household water storage [13]. Whenever possible, more than six larval habitats were examined per village. Mosquito larvae were transported to CREC, Cotonou and reared in an insectary under standard conditions (relative humidity  80 % ± 10 % and temperature 25 °C ± 2 °C). Adult mosquitoes were maintained with 10 % honey solution after emergence.

### Insecticide susceptibility test

Bioassays were carried out on 2–5 old females using deltamethrin at the diagnostic dose of 0.05 % according to standard WHO procedures [14]. The laboratory susceptible reference strain of *An. gambiae* (Kisumu) was used to check the quality of the impregnated paper. After 1 h of insecticide exposure, mosquitoes were transferred to holding tubes and fed with 10 % honey solution. Mortality was recorded 24 h post-exposure. For each test, mosquitoes were also exposed to untreated paper to assess natural (control) mortality and to keep a batch of non-exposed mosquitoes for biochemical and molecular studies. Abbott’s formula was used to correct the mortality when control mortality was between 5 and 20 %. After the test, legs were cut from control non-exposed mosquitoes for molecular determination and bodies were kept in RNA later at -20 °C for mRNA expression. Another batch was frozen at -80 °C for biochemical studies.

### Molecular and biochemical assays

Genomic DNA was extracted using cetyl trimethyl ammonium bromide (CTAB) 2 % method modified from Doyle 1987 [15]. Briefly, mosquitoes were ground in CTAB 2 % then heated in a water bath at 65° for 5 min. Chloroform was added to tubes, mixed by inversion, centrifuged and the upper phase transferred into another tube. DNA was precipitated with isopropanol and then washed once with 70 % cold ethanol. DNA was dried and suspended in distilled water. Species determination was performed by PCR [16]. The *1014 F* and *1014S kdr* mutations were detected by allelic discrimination Taqman assays as described by Bass [17] on field non-exposed females. Biochemical assay was used to quantify amounts of mixed function of oxidases (MFO), glutathione S-transferases (GST) and activities of non-specific esterase (NSE) using 30 female mosquitoes (non-exposed) for each village as described by Hemingway [18]. Each plate contained 10 unfed Kisumu adults used as the susceptible control. These biochemical tests were carried out on mosquitoes collected in June 2012 and June 2013 only due to insufficient sample size from others surveys.

### RNA extraction and reverse transcription quantitative PCR

Pools of each *Anopheles* species were used to determine the relative gene expression of CYP6M2, CYP6P3 and GSTD3 by qPCR using SYBR Green. Total mRNA was extracted from batches of five mosquitoes (stored in RNA later) using Isolate RNA micro kit (Bioline) according to the manufacturer’s instructions. Quantity and quality of mRNA were assessed using Nanodrop spectrophotometer (Nanodrop technologies). SuperScript III Reverse Transcriptase™ was used to synthesize first strand cDNA. The Kisumu was used as reference strain and the ribosomal gene RSP7 as housekeeping gene (shown to be consistent and with no differential expression between susceptible and resistant [19]). Three biological replicates were run for each sample and primers were designed on NCBI (http://www.ncbi.nlm.nih.gov/tools/primer-blast/) (Table 2). Real time PCR was run on Applied Bio systems ViiA7. Standard curves were generated using five times serially diluted cDNA sample to assess PCR efficiency. The PCR efficiency criterion was 100 ± 10 % for all of the genes and a single melting curve peak indicating specificity (Table 2). The cDNA was diluted 10-fold in this concentration that fitted within the dynamic range of each qPCR and stored at -20 °C.

**Table 2:** Primers used in RT-qPCR

Primer	Accession number	Primer sequence (5'–3')	PCR efficiency (%)
CYP6M2	VectorBase: AGAP008212	Fw: TCGGGATGTGTGCGTTCGGC	100
		Rv: TCGTGTCTCGCACCGCGTTC	
GSTD3	VectorBase: AGAP004382	Fw: CTAAGCTTAATCCGCAACATACCA	
		Rv: GTGTCATCCTTGCCGTACAC	93
RPS7	VectorBase: AGAP010592	Fw: ATTGCCGAGCGCCGCATTCT	
		Rv: GACGCGGATACGCTTGCCGA	100
CYP6P3	VectorBase: AGAP002865	Fw: TGTGATTGACGAAACCCTTCGGAAG	
		Rv: ATAGTCCACAGACGGTACGCGGG	97

### Data analysis

Mann-Whitney test implemented in R 2.15.2 software is used to compare (i) mosquito mortalities between villages and sampling periods; and (ii) levels of enzymatic activity between the lab reference strain and field mosquitoes. A linear mixed model with villages as random effect implemented in R 2.15.2 software was used to test the effect of surveys and villages on mortality. WHO criteria for discriminating individuals for susceptibility/resistance status were applied: 98–100 % mortality indicating susceptibility; 90–97 % suspected resistance and < 90 % mortality-confirmed resistance [14]. Spearman's correlation test was used to investigate (i) the link between *kdr* allele frequency of field mosquitoes and mortality; (ii) between *Anopheles* species and mortality; and (iii) the fold expression of cytochrome P450 and the mortality by survey. All differences were considered significant for *P*-value < 0.05. The relative expression of target genes were determined according to ΔΔCt methods described by Schmittgen & Livak [20]. QPCR data were analysed using simple statistical randomization tests implemented in REST 2009 software.

## Results

### Vector composition

**Fig. 1:**
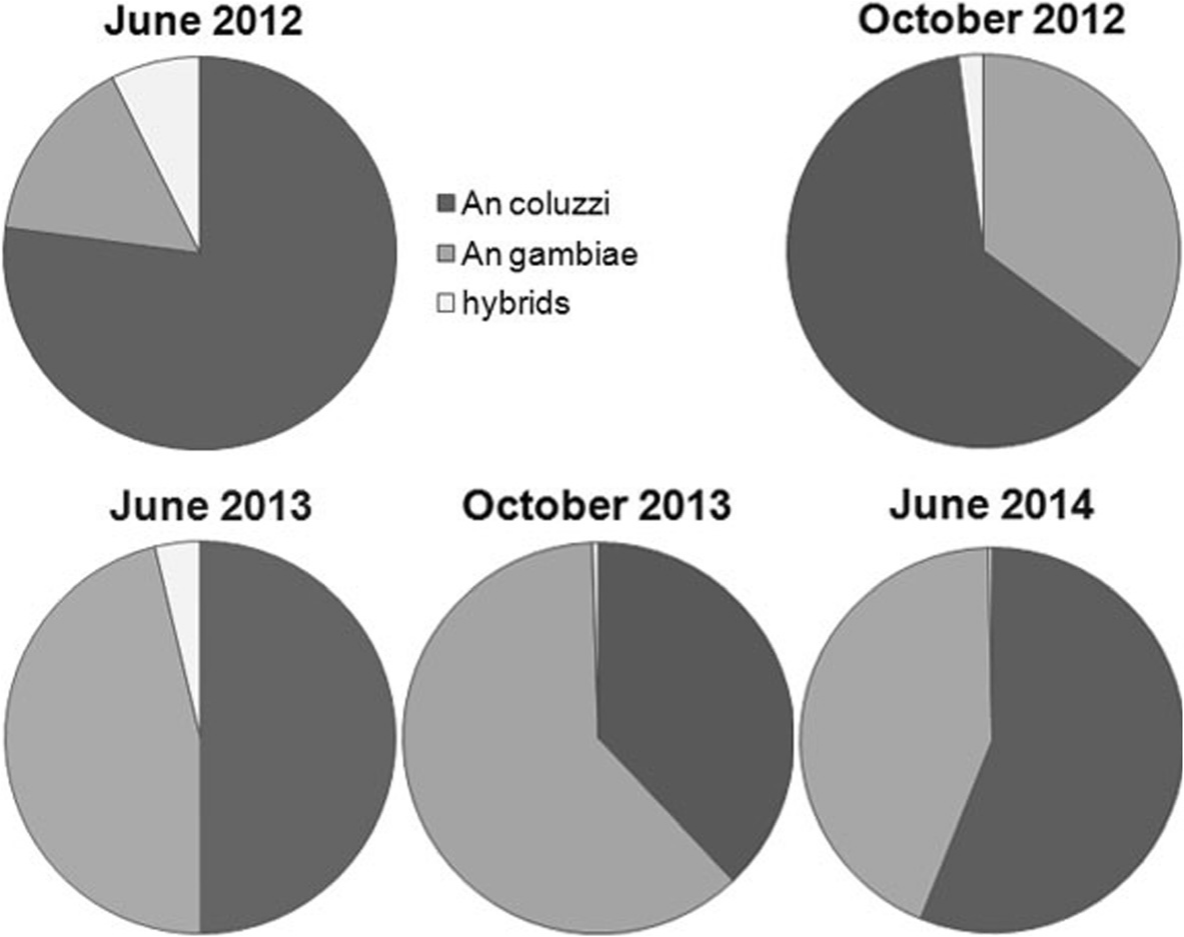
Proportions of *Anopheles gambiae* (*s.I.*) major species over sampling periods

*Anopheles coluzzii* and *An. gambiae* were found in sympatry in all villages. In June 2012, *An. coluzzii* was predominant in all sites (82 %) and *An. gambiae* and hybrids represented only 12 and 6 % of the species collected, respectively. Proportions of hybrids decreased over time to reach 0.3 % in June 2014. At the same time the proportion of *An. coluzzii* and *An. gambiae* fluctuated between 40 and 60 % (Fig. 1).

### Resistance status

**Fig. 2:**
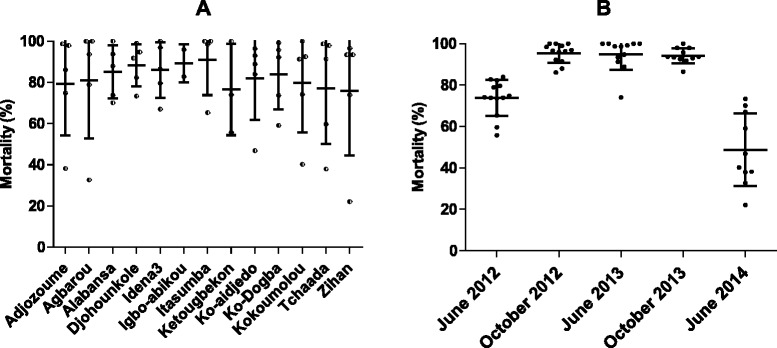
Phenotypic resistance to deltamethrin in *Anopheles gambiae* (*s.l*.) The scatter plot represents the mean mortalities with standard deviation based on 13 villages (**a**) and also according to surveys (**b**). The Mann-Whitney test showed significant time effect of sampling period on mosquito mortality while the variability between villages had no effect

Figure 2 shows the phenotypic resistance of malaria vectors to deltamethrin in thirteen villages during three years of follow-up. Mortality in controls never exceeded 5 % indicating that no contamination occurred during bioassays, then Abbott’s formula was not applied. Susceptibility tests were run on 3323; 1203; 827; 2955; and 1192 female *Anopheles* in June 2012, October 2012, June 2013, October 2013 and June 2014, respectively. There was no information available in three villages: Ketougbekon, Ko-aïdjedo (June 2013), Ko-aïdjedo, Kokoumolou (October 2013), Ketougbekon, Ko-aïdjedo, Kokoumolou (June 2014), due to the absence of *Anopheles* mosquitoes in all breeding habitats during the visit. The linear mixed model (AIC = 96, BIC = 57) showed that the sampling period had a significant effect on mortalities (“time-effect”, *P* < 0.001) but no effect of the villages (*P* = 0.9946) was recorded (Fig. 2).

In June 2012 and June 2014 (beginning of the rainy season) all *Anopheles* populations from the 13 villages were resistant to deltamethrin (mortalities < 90 %). From October 2012 to October 2013, mortalities exceeded 90 %, indicating that most of the *Anopheles* populations collected in the villages were suspected to be resistant. Three villages showed mortalities up to 97 % at the same period, indicating susceptibility. There was no strong correlation between mortality and proportions of both *Anopheles* species: *An.gambiae* (ρ = 0.34, CI _95%_ = 0.12–0.52, *P* = 0.008), *An. coluzzii* (ρ = -0.28, CI _95%_ = -0.510– -0.009, *P* = 0.030).

### *Kdr* mutations

In the study area, genotyping results showed that *1014 F kdr* allele frequency increased over time i.e. *f(1014 F)* was 0.67; 0.91; 0.92; 0.90 and 0.92 in June 2012, October 2012, June 2013, October 2013 and June 2014, respectively (Table 4). *Kdr* mutation was almost fixed in the study area and equally distributed between *An. coluzzii* and *An. gambiae*. *Anopheles* susceptibility to deltamethrin was not correlated with the *kdr* frequency over time (ρ = 0.146, CI _95%_ = 0.151–0.444, *P* = 0.336).

### Metabolic resistance

Activities of esterases, glutathione S-transferases and mixed function of oxidases were measured using mosquitoes collected in June 2012 and June 2013. Results showed a higher GST activity in field *Anopheles* in June 2012 and 2013 compared to the susceptible reference strain (Table 3). Similarly, the level of α-esterase activity of field mosquitoes was significantly higher than that at Kisumu in June 2012. Conversely, we did not report higher activity of P450 in field populations at the two surveys compared to the susceptible reference strain (*P* > 0.05).

**Table 3:** Detoxification enzyme activities of *Anopheles* in June 2012 and June 2013

	June 2012				June 2013			
	Mean *Anopheles*	Mean reference	FC	*P*-value	Mean *Anopheles*	Mean reference	FC	*P*-value
α-Naphthyl acetate	0.202 ± 0.08	0.118 ± 0.03	1.71	0.0001	0.140 ± 0.06	0.147 ± 0.08	0.95	0.463
β-Naphthyl acetate	0.141 ± 0.07	0.101 ± 0.04	1.39	0.0811	0.105 ± 0.10	0.117 ± 0.09	0.89	0.041
Oxydase (P450)	0.094 ± 0.05	0.090 ± 0.06	1.04	0.7617	0.038 ± 0.02	0.091 ± 0.03	0.41	0.0166
GST	0.264 ± 0.15	0.142 ± 0.03	1.86	<0.0001	0.521 ± 0.63	0.280 ± 0.65	1.86	0.001

Enzyme activities are expressed by mg of total proteins. Fold Change (FC) is the ratio of mean activity in *Anopheles*/mean activity in reference strain

**Fig. 3:**
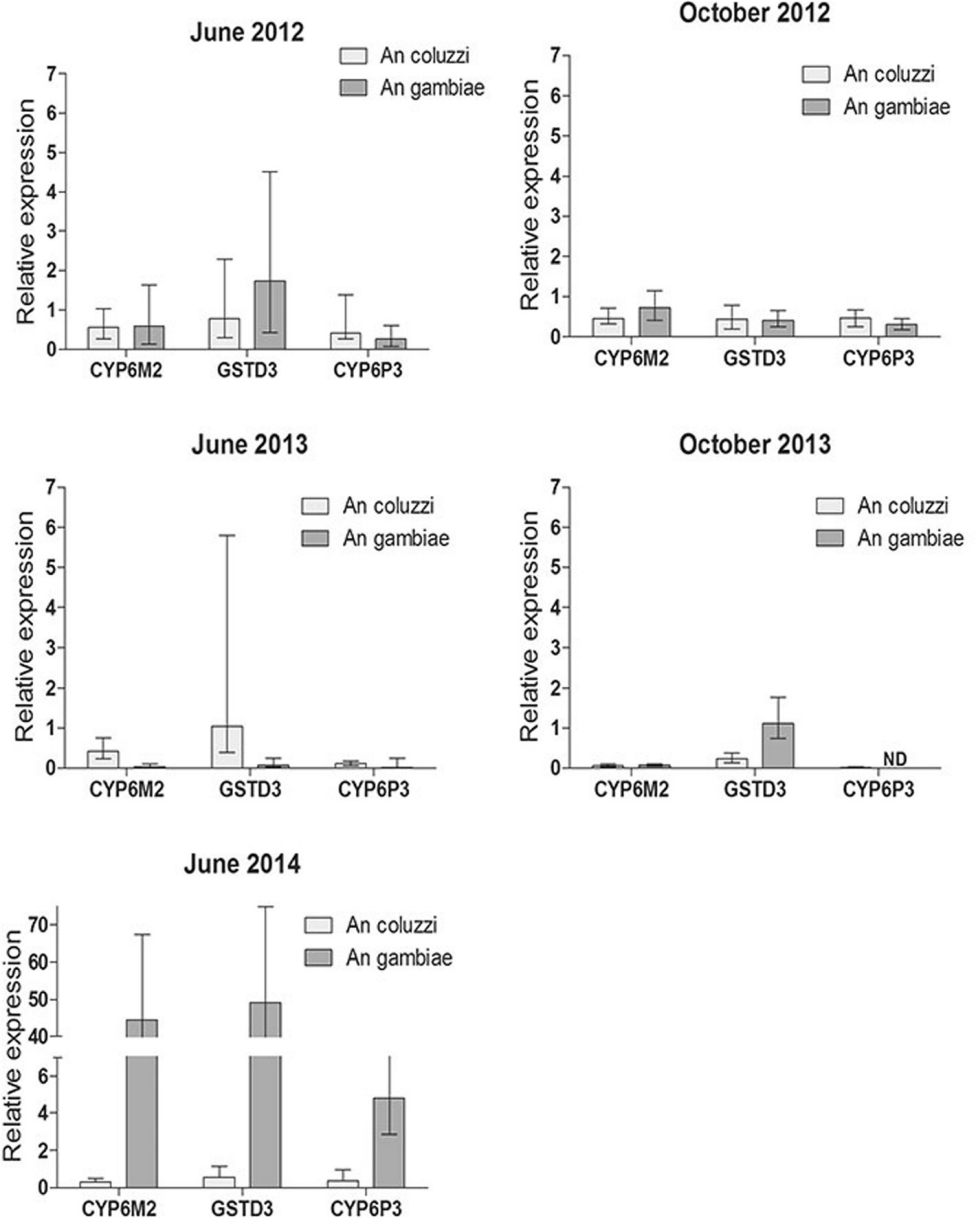
Relative expression of detoxification genes. ΔΔCt method was used for analysis. Bar charts represent the mean expression of three enzymes of field *Anopheles*. Error bars represent 95 % confidence interval. ND, no data

The expression of detoxification genes was also measured by RT-qPCR in both species (Fig. 3). Only *An. gambiae* showed upregulation of metabolic genes compared to reference strain. GSTD3 was upregulated in June 2012 and June 2014, the mean fold changes (FC) were 1.8 and 49.3, respectively (*P* < 0.001) (Fig. 3). CYP6P3 and CYP6M2 were upregulated in June 2014 during the beginning of the rainy season; the FC were 4.8 and 44.42, respectively (*P* < 0.001). These genes were mostly down regulated in October 2012 and October 2013, suggesting a relationship between the level of expression of some P450 markers and the resistance phenotype to deltamethrin.

## Discussion

This study addressed the dynamic of deltamethrin resistance in malaria vectors in the department of Plateau Benin, following a large scale implementation of malaria vector control tools. A combination of biological, biochemical and molecular assays were used to assess the frequency and mechanisms of pyrethroid-resistance in *An. gambiae* (*s.l.*) collected in 13 villages over three years. The results showed that *An. coluzzii* and *An. gambiae* were found in sympatry during all of the sampling periods but at various frequencies. A previous study demonstrated similar distribution of sibling species within *An. gambiae* (*s.l*.) complex in the study area [21].

Bioassay results showed significant variations of deltamethrin phenotypic resistance according to the surveys. *Anopheles* populations from the 13 villages were resistant to deltamethrin in June 2012 and June 2014 (< 90 % mortality) but mostly susceptible in October 2012 and 2013 (only 5 of 13 populations showed mortality < 90 %). This strong variation of phenotypic resistance in such a short period of time is difficult to explain knowing that environmental conditions did not change much between surveys (temperature 25 ± 2 °C and humidity 80 ± 10 %). The frequency of the *1014 F kdr* mutation was high in June 2012 in both species and did not increase much until June 2014. Consequently, it is unlikely that the *kdr* mutation alone contributed greatly to the phenotype observed. A previous study questioned the causal association between *kdr* genotype and pyrethroid resistance (especially for type II pyrethroids) and suggested that the *kdr* genotype may not necessarily be the best predictor of resistance in malaria vectors. The *1014S kdr* mutation originally from East Africa [22] was not detected in the present study. So far this mutation was found in Benin at a very low frequency in *An. arabiensis* [8].

In contrast, a significant increase in metabolic gene expression was reported in *An. gambiae* (*s.s*.) over the three-year follow-up. Overexpression of three metabolic genes, CYP6M2 (> 40-fold), GSTD3 (> 40-fold) and CYP6P3 (> 4-fold), were reported in June 2014, hence indicating a recent and strong selection pressure on this mosquito species. Interestingly, the high gene expression was associated with low mortality rates as measured by WHO bioassays. This finding supports the involvement of these metabolic markers in deltamethrin-resistant phenotype. Downregulation of these genes in June 2012 despite the presence of resistance phenotype may be explained by the low number of *An. gambiae* collected in that survey (Table 4) and/or by the involvement of other (non-detectable) metabolic detoxification genes. The differential expression of metabolic markers between *An. gambiae* and *An. coluzzi* has been reported in Benin [23] and may reflect differential exposure to insecticides/xenobiotics at larval and/or adult stage.

**Table 4:** *Kdr* allelic frequencies in *An. coluzzi* and *An. gambiae. f(1014)F* was determined by *qPCR*. N represents the number of alleles tested

Villages	June 2012				October 2012				June 2013				October 2013				June 2014			
	*An. coluzzi*		*An. gambiae*		*An. coluzzi*		*An. gambiae*		*An. coluzzi*		*An. gambiae*		*An. coluzzi*		*An. gambiae*		*An. coluzzi*		*An. gambiae*	
	*f(1014)F*	N	*f(1014)F*	N	*f(1014)F*	N	*f(1014)F*	N	*f(1014)F*	N	*f(1014)F*	N	*f(1014)F*	N	*f(1014)F*	N	*f(1014)F*	N	*f(1014)F*	N
Adjozoume	0.65 (0.51–0.76)	62	0.7 (0.34–0.93)	10	1 (0.39–1)	4	1 (0.90–1)	38	1 (0.83–1)	20	1 (0.95–1)	72	−	−	1 (0.92–1)	48	−	−	1 (0.92–1)	50
Agbarou	0.73 (0.56–0.85)	40	0.75 (0.42–0.94)	12	1 (0.39–1)	4	1 (0.91–1)	42	0.8 (0.61–0.92)	30	1 (0.83–1)	20	0.83 (0.71–0.90)	70	0.99 (0.93–0.99)	82	0.88 (0.78–0.93)	82	1 (0.91–1)	40
Alabansa	0.73 (0.59–0.83)	62	0.68 (0.47–0.84)	28	0.82 (0.65–0.93)	34	1 (0.94–1)	68	0.89 (0.75–0.97)	38	0.97 (0.89–0,99)	66	0.92 (0.77–0.98)	36	0.99 (0.96–0.99)	170	0.89 (0.76–0.96)	46	1 (0.93–1)	54
Djohounkole	0.68 (0.58–0.77)	92	1 (0.54–1)	6	0.89 (0.65–0.98)	18	0.90 (0.68–0,98)	20	0.84 (0.71–0.92)	56	0.91 (0.74–0.98)	32	0.94 (0.84–0.98)	62	1 (0.95–1)	88	0.83 (0.70–0.91)	58	1 (0.73–1)	12
Idena3	0.68 (0.58–0.76)	118	0.75 (0.34–0,96)	8	0.88 (0.47–0,99)	8	0.99 (0.94–0,99)	106	0.93 (0.66–0.99)	14	1 (0.87–1)	28	0.71 (0.41–0.91)	14	1 (0.97–1)	182	0.87 (0.75–0.94)	54	0.97 (0.88–0.99)	46
Igbo-abikou	0.73 (0.54–0.87)	30	0.83 (0.35–0,99)	6	0.85 (0.65–0.95)	26	0.98 (0.93–0.99)	104	0.75 (0.34–0.96)	8	1 (0.95–1)	90	1 (0.76–1)	14	1 (0.97–1)	130	0.78 (0.65–0.87)	60	1 (0.91–1)	42
Itasumba	0.66 (0.50–0.79)	44	0.5 (0.19–0.98)	2	0.78 (0.69–0.86)	96	1 (0.63–1)	8	0.79 (0.69–0.86)	100	1 (0.15–1)	2	0.82 (0.75–0.86)	202	−	−	0.71 (0.60–0.80)	80	_	−
Ketougbekon	0.65 (0.50–0.78)	52	0.83 (0.35–0.99)	6	0.84 (0.71–0.92)	56	0.96 (0.87–0.99)	68	0,88 (0.71–0.96)	32	1 (0.81–1)	18,00	0.91 (0.84–0.95)	122	0.84 (0.73–0.91)	74	0.90 (0.80–0.96)	72	0.86 (0.67–0.95)	28
Ko-aidjedo	0.62 (0.40–0.79)	26	0.42 (0.15–0.72)	12	0.5 (0.19–1)	2	0.96 (0.89–0.99)	84	−	−	−	−	−	−	−	−	−	−	−	−
Ko-Dogba	0.64 (0.52–0.75)	76	0.73 (0.56–0.85)	40	1 (0.39–1)	4	0.92 (0.83–0.96)	84	0.83 (0.51–0.97)	12	1 (0.84–1)	22	0.5 (0.06–0.93)	4	0.97 (0.90–0.99)	90	−	−	−	−
Kokoumolou	0.60 (0.46–0.71)	62	0.79 (0.49–0.95)	14	0.87 (0.79–0,92)	108	1 (0.86–1)	26	0.9 (0.68–0.98)	20	1 (0.81–1)	18	−	−	−	−	−	−	−	−
Tchaada	0.84 (0.74–0.90)	98	−	−	0.94 (0.80–0.99)	34	0.92 (0.77–0.98)	36	0.86 (0.71–0.94)	42	0.88 (0.47–0.99)	8	0.92 (0.84–0.96)	92	0.98 (0.87–0.99)	68	0.93 (0.79–0.98)	40	1 (0.63–1)	8
Zihan	0.36 (0.23–0.49)	58	0.63 (0.24–0.91)	8	0.89 (0.80–0.94)	82	0.93 (0.79–0.98)	40	0.84 (0.72–0.91)	68	1 (0.83–1)	20	0.78 (0.67–0.86)	78	1 (0.94–1)	68	1 (0.69–1)	10	1 (0.91–1)	40

CYP6M2 and CYP6P3 are regularly associated with pyrethroid resistance [24] and have been validated as pyrethroid metabolizers [25, 26]. No correlation was noted, however, between the oxidase activity and P450 gene expression. This can be explained by a lack of sensitivity and specificity of biochemical assays that employ generic heme peroxidase assays that are recognized by many members of the enzyme family [25]. The use of mixed populations (*An. gambiae* and *An. coluzzii*) during biochemical tests may have also affected the outcomes. Overexpression of CYP6M2 and CYP6P3 is widespread in West Africa including Benin, Nigeria [19], Ghana [26] and should be routinely monitored by national malaria control programmes. In addition, GSTD3 was found up regulated in *Anopheles* in June 2012 and 2014 hence correlating results of both, the biochemical test and bioassays. GSTD3 was found upregulated in DDT-resistant *An. arabiensis* and *An. gambiae* in Burkina Faso and Benin, respectively [8]. GSTs are regularly found overexpressed in many pyrethroid-resistant mosquitoes [27, 28]. Some studies suggested their potential role against oxidative stress [29] and in pyrethroid sequestration [30]. Although the role of mosquito GSTs in pyrethroid resistance is likely, understanding the underlying mechanisms requires further investigations.

In Benin, the use of pesticides for both vector control and agricultural practices (cotton crops and vegetable farms) are known to be a major source of selection pressure on malaria vectors [31–33]. For example, a randomized controlled trial conducted in southern Benin showed an increase in the *kdr* frequency from 20 to 80 % in *An. gambiae* eighteen months after the distribution of LLIN at community level [33]. We suspect that the increased coverage of deltamethrin-LLINs in 2014 in the study area (80 %, NMCP) has contributed to selection for pyrethroid-resistance metabolic markers. The role of agricultural practices in the selection of insecticide resistance in *An. gambiae* remains unknown. No significant changes in land use and agricultural practices were observed during the three-year follow-up but we could not record the type and amount of insecticides used by native farmers for crop protection. Similarly, the larval exposure to various xenobiotics (e.g. heavy metals, oils, fungicides, pollutants) that are known to modulate/enhance the metabolic detoxification profile of mosquitoes could not be investigated [34, 35]. Clearly, much work has to be done to address the environmental factors contributing to resistance selection in malaria vectors in Benin.

The present study was part of a multidisciplinary project funded by the Bill & Melinda Gates Foundation that aims at addressing whether insecticide resistance can impact on the effectiveness of malaria vector control tools in Africa. Up to now the results of the first year follow-up did show substantial impact of insecticide resistance on the efficacy of LLINs in southern Benin [36] but analyses of years 2 and 3 are still ongoing.

## Conclusions

This study monitored the levels and mechanisms of deltamethrin resistance in major malaria vectors in the Plateau department, Benin, after a three-year implementation of malaria vector control. The results showed that resistance to deltamethrin in malaria vectors was widespread and multifactorial. We also suspect that the increase in deltamethrin resistance between 2012 and 2014 resulted from an increased expression of metabolic detoxification genes (CYP6M2 and CYP6P3) rather than *kdr* mutations. It is now urgent to evaluate further the impact of metabolic resistance on the efficacy of vector control interventions using pyrethroid insecticides.

## Abbreviations

CTAB, Cetyl Trimethyl Ammonium Bromide; DDT, Dichlorodiphényltrichloroéthane; GST, Gluthatione S-transferase; IRS, Indoor Residual Spraying; *Kdr*, Knockdown resistance; LLIN, Long Lasting Insecticide Nets; MFO, Mixed Function of Oxidase; NMCP, National Malaria Control Program; NSE, Non-Specific Esterase; WHO, World Health Organization
